# Association between the Weight-Adjusted Waist Index and Serum Uric Acid: A Cross-Sectional Study

**DOI:** 10.1155/2023/8215866

**Published:** 2023-07-28

**Authors:** Huan Li, Guowei Fang, Chengcheng Huang, Wenrong An, Xiaohan Bai, Yanqin Huang

**Affiliations:** ^1^First Clinical Medical College, Shandong University of Traditional Chinese Medicine, Jinan 250014, China; ^2^Department of Endocrinology, Affiliated Hospital of Shandong University of Traditional Chinese Medicine, Jinan 250014, China; ^3^Department of Geriatrics, Second Affiliated Hospital of Shandong University of Traditional Chinese Medicine, Jinan 250014, China; ^4^Department of Traditional Chinese Medicine, The 960th Hospital of the PLA Joint Logistics Support Force, Jinan 250014, China

## Abstract

**Background:**

Serum uric acid (SUA) was closely related to body metabolism. This study aimed to investigate the relationship between the adult weight-adjusted waist index (WWI) and SUA.

**Methods:**

In the National Health and Nutrition Examination Survey (NHANES) from 2011 to 2020, 6494 eligible participants aged ≥20  were included. The multivariate logistic regression model was used to test the correlation between WWI and SUA. At the same time, subgroup analysis was carried out by using multivariate logistic regression according to age, sex, and race. Then, the fitting smooth curve was applied to solve the association between WWI and SUA. Finally, the recursive algorithm was used to calculate the inflection point in the nonlinear relationship, and the two-stage piecewise linear regression model was used to analyze the relationship between WWI and SUA on both sides of the inflection point.

**Results:**

In all the 6494 participants, through the fully adjusted model, this study found that there was a positive correlation between WWI and SUA (*β* = 5.64; 95% CI: 2.62 and 8.66). In addition, this positive correlation still had certain statistical significance in the subgroup analysis stratified by sex, age, and race. Our research team found a significant positive correlation between the WWI and SUA in females, but the correlation was not significant in males. We also found a small inverted U-shaped curve between the WWI and SUA in men when we stratified the sex subgroups. The small inflection point was determined to be 11.5 cm/√ kg. In racial subgroup analysis, we also found a U-shaped relationship between the WWI and SUA in non-Hispanic White and other race/ethnicity (the inflection point was 11.08 cm/√ kg and 12.14 cm/√ kg, respectively).

**Conclusion:**

This study showed that the WWI was a newly developed and new predictor of centripetal obesity independent of body weight and there was a positive correlation between the WWI and SUA.

## 1. Introduction

Obesity was brought on by an increase in the quantity and/or size of fat cells as well as an improper distribution of fat. Obesity often increases people's chances of developing various diseases, shortens life expectancy, and lowers the quality of life. Obesity had gradually escalated in importance as a public health issue [1]. Globally, the prevalence of obesity has been steadily rising in recent years. By 2025, it is predicted that 21% of adult women and 18% of adult men will be obese, with more than 6% of men and 9% of women developing severe obesity [2]. The distinction between muscle mass and fat volume is now problematic for classic obesity markers such as body mass index (BMI), a body shape index (ABSI), and waist circumference (WC), making BMI incapable of identifying normal-weight obesity (NWO). As a result, these anticipated measures of obesity cannot adequately reflect the connection between fat and unfavorable health outcomes. The weight-adjusted waist index (WWI), which Park et al. proposed in 2018 and which had a positive correlation with fat content [3], could highlight the issue of central obesity. Uric acid was produced by the metabolism of purine compounds in the body in the liver, mainly excreted by the kidneys. Excessive uric acid production and an imbalance in the body's excretion were the two main causes of hyperuricemia [4]. According to research, excessive fat storage in obese persons will impair their liver, which will cause uric acid levels to rise [5]. In addition, an increase in hepatic and peripheral fat production brought on by a rise in SUA in the body would result in obesity, further demonstrating the relationship between obesity and hyperuricemia [6]. Serum uric acid (SUA) had recently been linked to obesity in numerous studies [7], but the majority of these studies used BMI as their primary indicator of obesity. According to several studies, uric acid and BMI were highly positively correlated [8]. The relationship between WWI and urinary albumin has now been studied by Zheng Qin et al., who found a positive correlation between the two. They also found that WWI, a newly developed obesity index, was more closely related to BMI and WC and that it can be used as a measurement index to predict urinary albumin. Previous research had not looked into the connection between SUA and WWI [9].

The authors of this study contend that by assessing the connection between WWI and SUA, hyperuricemia can be efficiently avoided and its prognosis enhanced. As a result, the purpose of this study is to investigate how WWI and SUA are related using data from the nationally representative NHANES. Due to the contrasts between SUA and WWI in terms of gender, age, race, etc., for the purpose of demonstrating the relationship in several subgroups, this study carried out subgroup analysis in accordance with the STROBE guidelines [10].

## 2. Materials and Methods

### 2.1. Study Population

The data of this cross-sectional study were from the National Health and Nutrition Examination Survey (NHANES) from 2011 to 2020. NHANES is a study conducted by the National Center for Health Statistics (NCHS) to evaluate the health and nutritional status of representative samples of the population in the United States. The research was carried out by using multistage, hierarchical, and clustering probability design and was monitored and investigated continuously. All procedures of the NHANES were reviewed and approved by the Research Ethics Review Committee of the National Health Statistics Center, and the written informed consent of all participants was obtained in the annual survey. Our current research does not include any identification materials of people. Therefore, this study does not require further ethical review, and all data in this study can be downloaded from the official website of the NHANES.

In this study, 45462 participants were enrolled in the NHANES cycle from 2011 to 2020. Our inclusion criteria were 26280 subjects aged over 20. 5243 subjects with the incomplete WWI and 2429 subjects with incomplete SUA data were excluded, and 2590 cancer patients were excluded. Finally, subjects with complete WWI, SUA data, and other covariate data were included in our follow-up analysis (*n* = 6494).

### 2.2. Evaluation of the Weight-Adjusted Waist Index

The WWI was a human measurement index for waist circumference (cm) and body weight (kg), mainly used to estimate whether the body was central to obesity. The higher the WWI ratio, the greater the degree of central obesity. The data about waist circumference and weight measured by the body were collected by trained health technicians in the mobile examination center (MEC). The WWI of each participant in this study was calculated by dividing the waist circumference in centimeters by the square root of the weight in kilograms. In this analysis, we regard WWI as a continuous variable and group participants according to the quartile of WWI for further analysis. In our study, WWI was designed as an exposure variable.

### 2.3. Evaluation of Serum Uric Acid

The data of serum uric acid were detected in the serum samples of the NHANES participants obtained from MEC. The concentration of SUA was determined by using the timed endpoint method. The concentration of SUA was calculated by monitoring the absorbance change of the colored product produced by the reaction of hydrogen peroxide produced by the oxidation of uric acid with 4-amino antipyrine catalyzed by 3,5-dichloro-2-hydroxybenzenesulfonate. In our analysis, SUA was designed as an outcome variable.

### 2.4. Evaluation of Other Covariates

Other variables in this study included age, gender, race, the ratio of family income to poverty, education level, body mass index (BMI), the intake of energy and nutrition in the diet, systolic blood pressure (SBP), diastolic blood pressure (DBP), glycosylated hemoglobin, urinary albumin, urinary creatinine, urinary albumin-creatinine ratio, low-density lipoprotein cholesterol (LDL-C), high-density lipoprotein cholesterol (HDL-C), triglyceride (TG), total cholesterol (TCHO), smoking status (whether you have smoked at least 100 cigarettes in your life), drinking status (used to drink 4/5 or more per day), and sedentary time. The criteria for selecting covariates were based on the previously published studies and variables [11]. The data used in this study can be found on the NHANES website.

### 2.5. Statistical Analysis

**Figure 1:**
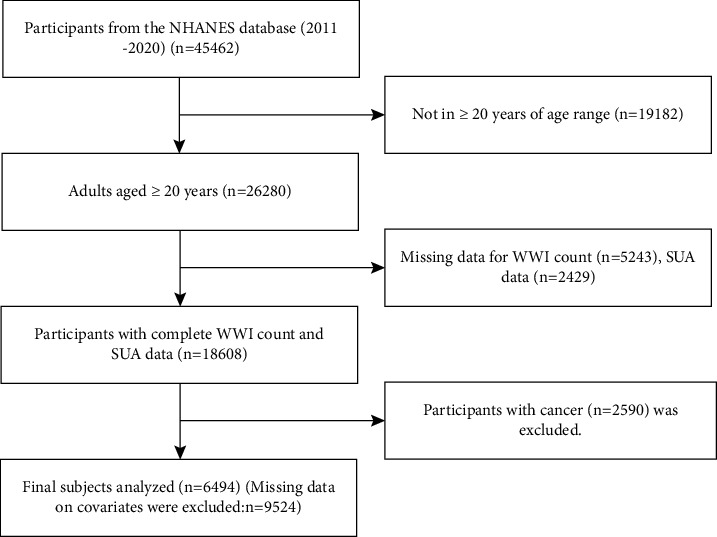
Flowchart of participant selection. NHANES, National Health and Nutrition Examination Survey; WWI, weight-adjusted waist index; SUA, serum uric acid.

According to the NHANES analysis guidelines, the baseline data in this study were expressed in terms of the mean value ± standard deviation (SD) of continuous variables and the frequency (percentage) of categorical variables. This study used a multivariate linear regression model to estimate the influence of *β*s and its 95% confidence interval on the relationship between WWI and SUA. WWI was analyzed as a continuous variable and categorical variable (quartile), and the baseline characteristic difference of the WWI quartile was compared using the one-way analysis of variance (ANOVA) test of a continuous variable and the chi-square test of categorical variable. No covariates were adjusted in the model 1 analysis. Model 2 analysis was adjusted for age, sex, and race. Based on the analysis of model 2, model 3 also aims at the intake of energy and nutrition (energy, protein, carbohydrate, dietary fiber, and fat) in the diet, education level, the proportion of family income to poverty, BMI, DBP, SBP, glycosylated hemoglobin, urinary albumin, urinary creatinine, urinary albumin-creatinine ratio, LDL-C, HDL-C, TG, TCHO, and health-related behaviors (smoking, drinking, and sedentary time). In addition, subgroup analysis was conducted by the stratified multiple regression method according to sex (female or male), age (≥20, <40 or ≥40, <60 or ≥60, <80 or ≥80 years old), race (Mexican American or other Hispanic or non-Hispanic White or non-Hispanic Black or other race/ethnicity). In addition, this study solved the relationship between WWI and SUA by using smoothing and generalized additive models. When nonlinearity was detected, a recursive algorithm was used to calculate the inflection point. The flowchart of this study was shown in Figure 1.

## 3. Result

### 3.1. Baseline Characteristics of the Participants

The baseline characteristics of all the participants were shown in Table 1. A total of 6494 participants (3419 males and 3075 females) were included in the study, with an average age of 47.46 ± 16.78 years. All variables and indicators in this study were significantly different from the baseline characteristics of the WWI quartile. Compared with other subgroups, the highest quartile of the WWI participants was more likely to be middle-aged people, female, non-Hispanic White, less energy and dietary intake, higher BMI, lower family income and poverty ratio, higher level of education, lower high-density lipoprotein cholesterol, higher triglycerides, higher urinary albumin, lower urinary creatinine, higher urinary albumin/creatinine ratio, higher glycosylated hemoglobin, higher systolic blood pressure, preference for smoking, and long sitting time.

### 3.2. Relationship between WWI and SUA

Table 2 showed the multiple regression analysis results of WWI and SUA. The results of this study showed that the higher WWI was related to the increased possibility of SUA. A positive correlation between WWI and SUA was detected in the unadjusted model 1 (*β* = 12.97, 95% CI: 10.53 and 15.41, and *P* < 0.0001). After adjusting for confounding factors (age, sex, and race), this positive correlation still existed in model 2 (*β* = 25.38, 95% CI: 22.83 and 27.94, and *P* < 0.0001), and after fully adjusting the covariates, we found that this positive correlation still existed in model 3 (*β* = 5.64, 95% CI: 2.62 and 8.66, and *P* = 0.0003). When we converted the WWI from a continuous variable to a categorical variable (quartile), we found that the association between WWI and SUA was still statistically significant at the higher WWI. After converting the WWI from a continuous variable to a categorical variable (quartiles), individuals in the highest quartile had a 7.93 *μ*mol/L higher SUA than those in the lowest WWI quartile after adjusting confounding variables (model 3, *β* = 7.93, 95% CI: 1.42 and 14.45). In addition, the results of this study using smooth curve fitting and a generalized additive model showed that there was a linear relationship between the WWI and the risk of SUA.

In the subgroup analysis by sex, age, and race/ethnicity reported in Table 2, the positive correlation of the WWI with SUA remained in male (*β* = 8.16, 95% CI: 3.10 and 13.23, and *P* = 0.0016), female (*β* = 5.67, 95% CI: 1.96 and 9.38, and *P* = 0.0028), age ranged from ≥20 to <40 (*β* = 7.05, 95% CI: 2.59 and 11.51, and *P* = 0.0020), age ranged from ≥40 to <60 (*β* = 8.24, 95% CI: 3.34 and 13.14, and *P* = 0.0010), as well as in non-Hispanic Black (*β* = 7.26, 95% CI: 0.83 and 13.70, and *P* = 0.0272) and other race/ethnicity (*β* = 11.88, 95% CI: 3.26 and 20.51, and *P* = 0.0071), but not in Mexican American, other Hispanic, non-Hispanic White, and people with an age ranged from ≥60.

In this study, our team found a positive correlation between WWI and SUA in the face of age stratification. When this study stratified the gender subgroups, we found a significant positive correlation between WWI and SUA in the female population, while the positive correlation between the two was not significant in the male population. On the contrary, there was a small inverted U-shaped curve between WWI and SUA in the male population. The small inflection point was determined to be 11.5 cm/√ kg by using a two-stage linear regression model. When WWI < 11.5 cm/√ kg, each increase of 1 cm/√ kg in WWI will result in an increase of 11.41 *μ*mol/L in SUA (95% CI: 5.62 and 17.20). In contrast, for WWI > 11.5 cm/√ kg, for every 1 cm/√ kg increase in WWI, SUA will decrease by 5.09 *μ*mol/L (95% CI: −17.63 and 7.45).

**Figure 2:**
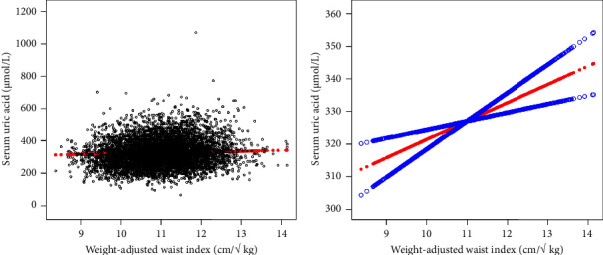
The association between WWI and SUA. (a) Each black point represents a sample. (b) The solid red line represents the smooth curve fit between variables. Blue bands represent the 95% of confidence interval from the fit. Age, sex, race/ethnicity, education level, the ratio of family income to poverty, BMI, energy intake, protein intake, carbohydrate intake, dietary fiber intake, total fat intake, LDL-C, HDL-C, TG, TCHO, urine albumin, urine creatinine, urine albumin creatinine ratio, HbA1c, SBP, DBP, heavy alcohol consumption, smoking status, and minutes sedentary activity were adjusted.

**Figure 3:**
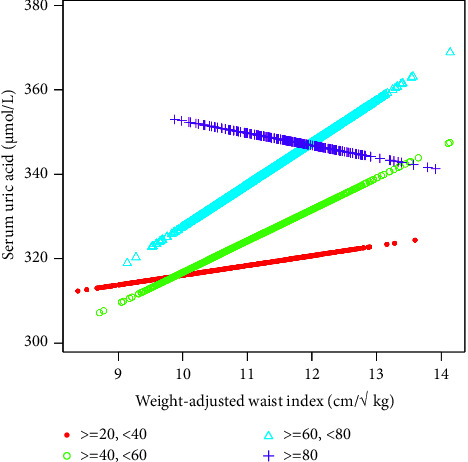
The association between WWI and SUA stratified by age. Sex, race/ethnicity, education level, the ratio of family income to poverty, BMI, energy intake, protein intake, carbohydrate intake, dietary fiber intake, total fat intake, LDL-C, HDL-C, TG, TCHO, urine albumin, urine creatinine, urine albumin creatinine ratio, HbA1c, SBP, DBP, heavy alcohol consumption, smoking status, and minutes sedentary activity were adjusted.

**Figure 4:**
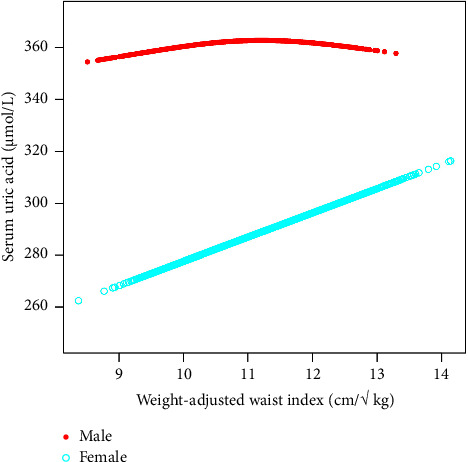
The association between WWI and SUA stratified by sex. Age, race/ethnicity, education level, the ratio of family income to poverty, BMI, energy intake, protein intake, carbohydrate intake, dietary fiber intake, total fat intake, LDL-C, HDL-C, TG, TCHO, urine albumin, urine creatinine, urine albumin creatinine ratio, HbA1c, SBP, DBP, heavy alcohol consumption, smoking status, and minutes sedentary activity were adjusted.

**Figure 5:**
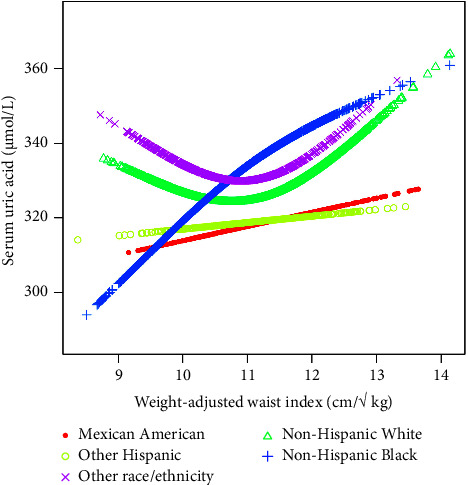
The association between WWI and SUA stratified by race/ethnicity. Age, sex, education level, the ratio of family income to poverty, BMI, energy intake, protein intake, carbohydrate intake, dietary fiber intake, total fat intake, LDL-C, HDL-C, TG, TCHO, urine albumin, urine creatinine, urine albumin creatinine ratio, HbA1c, SBP, DBP, heavy alcohol consumption, smoking status, and minutes sedentary activity were adjusted.

We found a U-shaped relationship between the WWI and SUA of non-Hispanic White and other race/ethnicity in the stratification of the race status, and the inflection point was determined using a two-stage linear regression model. In the non-Hispanic White, the inflection point was 11.08 cm/√ kg. For WWI < 11.08 cm/√ kg, each increase of 1 cm/√ kg in the WWI will result in a decrease of 7.08 *μ*mol/L in SUA (95% CI: −14.39 and 0.23). In contrast, for the WWI greater than 11.08 cm/√ kg, each increase of 1 cm/√ kg in the WWI will result in an increase of 10.73 *μ*mol/L in SUA (95% CI: 3.78 and 17.68). In other races/efficiency, the inflection point was 12.14 cm/√ kg. When WWI < 12.14 cm/√ kg, each increase of 1 cm/√ kg in the WWI will result in an increase of 7.79 *μ*mol/L in SUA (95% CI: −1.26 and 16.84). In contrast, for WWI > 12.14 cm/√ kg, for every 1 cm/√ kg increase in the WWI, SUA will increase by 87.10 *μ*mol/L (95% CI: 33.93 and 140.27). The results of threshold analysis in the subgroup stratification of gender and race were shown in Tables 3–5, respectively. Smooth curve fittings and generalized additive models used to characterize the nonlinear relationship between WWI and SUA were shown in Figures 2–5.

## 4. Discussion

This study was the first cross-sectional study to evaluate the relationship between WWI and SUA. This study showed that in all three models of analyzing NHANES 2011–2020 data, our team found that WWI has a significant positive correlation with SUA of adults over the age of 20 and remains stable. In the past studies, some epidemiological studies had shown that there was a positive correlation between obesity and SUA [12–14]. In 2021, Nam Hoon Kim et al. showed that in the cross-sectional sample of 602 participants aged ≥65 , by evaluating the correlation between WWI and muscle and fat mass, it was found that WWI was positively correlated with fat mass and negatively correlated with muscle mass in the elderly [15], which also coincided with the results of the correlation between WWI and SUA in terms of age stratification. In addition, Andrea Ungar et al. found that the relationship between SUA and mortality of the oldest participants was J-shaped [16], which not only revealed the considerable difference between SUA and age but also clarified that the SUA level of the older participants in this study gradually returned to a stable state. However, the special mechanism between WWI and SUA at different age stages also hopes to be explained in the future. Compared with normal-weight individuals, SUA secretion in obese individuals was more [17]. SUA could increase the fat stored in the body, thus increasing the concentration of triglycerides [18] and predicting weight gain [19].

The relationship between obesity and SUA could be defined by way of the following pathophysiological mechanisms. First of all, obesity or too much fat in the body will produce insulin resistance, stimulate the synthesis of triglycerides [13], increase the production of xanthine oxidoreductase in obesity [20], and stimulate the secretion of uric acid in the adipose tissue by regulating human metabolism and lead to the imbalance of uric acid metabolism and even develop into hyperuricemia. Second, elevated SUA concentration could cause damage to pancreatic islet *β* cells by inhibiting the availability of nitric oxide in the body and reducing glucose uptake [21] and inducing and aggravating insulin resistance [22]. After insulin resistance, a large amount of insulin accumulated in the body will inhibit fat decomposition and promote the production of the liver and peripheral fat, leading to obesity [23]. Insulin resistance caused by obesity will activate the renin-angiotensin-aldosterone system of the body, increase the damage to renal function, and also affect the regulation of uric acid levels by renal function [24]. According to their inseparable physiological and pathological mechanisms, a vicious circle of hyperuricemia and obesity was a step by step-shaped in the body. More research had proven that SUA had a tremendous correlation with inflammatory markers such as C-reactive protein and interleukin-6, which was extra intently associated with metabolic ailments (such as obesity) [25]. Early studies had shown that estrogen could downregulate the expression of urate reabsorption transporters, thereby increasing uric acid excretion and reducing the concentration of SUA in the body [26].

In this study, our team found that the linear relationship between WWI and SUA showed a significant positive correlation between men and women. With the increase of WWI, the SUA of women increases steadily, while the increase of SUA of men was not significant. The relationship between WWI and SUA of men (turning point: 11.50 cm/√ kg) follows a small inverted U-shaped curve, which might additionally be associated with the fat distributions in the body. In addition, studies had shown that the relationship between women's SUA concentration and the incidence rate of cardiovascular disease and renal insufficiency was stronger than that of men [27, 28] and that the high SUA level was more related to the risk of obesity in women [29], which could be seen that different uric acid levels had different effects on WWI and indirectly indicated that women might be more vulnerable to the impact of different SUA concentrations. This coincides with the results of subgroup stratified analysis based on gender in this study. In the analysis of this study, age, sex, race, and other variables were adjusted for multiple regression and lifestyle (sedentary time, drinking, and smoking), dietary energy intake, education level, blood pressure, blood glucose, blood lipid level, and renal function levels were also considered. In general, it was significant to clarify the difference between SUA concentration and obesity in men and women. In the future, when implementing measures to prevent the rise of SUA levels caused by obesity, we should take into account the differences due to gender differences and we can apply this finding to clinical research. Our research had some advantages and limitations. First of all, our study was based on a large-scale nationwide data, which was applicable to the general population in the United States. All participants had complete data on WWI and SUA. After adjusting the regression analysis for all covariates, we found that WWI and SUA do have a significant positive correlation. Then, this study conducted a subgroup analysis to further explain the different association patterns of WWI and SUA in gender, age, and race, which also confirmed the age, gender, and race differences that were easily ignored in clinical practice. The WWI in our study was a relatively new indicator to measure obesity. Compared with the traditional BMI-based method, it has certain exploration. Of course, the limitations of this study were also noteworthy. First, the essence of a cross-sectional study was to determine the outcome factors and exposure factors at the same time, which cannot infer the causal relationship between WWI and SUA. Second, the data we collected were not the most comprehensive and the data volume was not the largest. We still need further large-sample prospective research to explore this causal relationship. Third, although our researchers had adjusted potential covariates, such as dietary and nutritional status [30], SBP, DBP, glycosylated hemoglobin, blood lipid level, renal function status, smoking and drinking status, and physical activity level, we cannot completely eliminate the risk of bias caused by other potential confounding factors. Although there were some limitations, the results of this study were helpful to public health and also confirmed that WWI, as a newly developed index for predicting central obesity, had clinical significance not only in cardiovascular diseases but also in exploring the relationship between obesity and uric acid.

## 5. Conclusion

This study showed that WWI was a newly developed and new predictor of centripetal obesity independent of body weight and there was a positive correlation between WWI and SUA.

## Figures and Tables

**Table 1:** Baseline characteristics of all the participants were stratified according to the quartile of the weight-adjusted waist index (WWI).

Variables# WWI quartile	Total	Quintile categories of WWI (cm/√ kg)				*P* value
		Q1 (8.38–10.41)	Q2 (10.41–10.98)	Q3 (10.98–11.56)	Q4 (11.56–14.14)	
Participants	6494	1624	1623	1623	1624	
SUA (*μ*mol/L)	327.00 ± 84.37	312.41 ± 78.96	321.97 ± 80.75	334.88 ± 84.40	338.75 ± 90.39	<0.001
Age (years)	47.46 ± 16.78	36.58 ± 13.38	44.88 ± 14.96	51.27 ± 15.55	57.09 ± 15.78	<0.001
Sex (%)						<0.001
Male	3419 (52.65%)	1028 (63.30%)	931 (57.36%)	856 (52.74%)	604 (37.19%)	
Female	3075 (47.35%)	596 (36.70%)	692 (42.64%)	767 (47.26%)	1020 (62.81%)	
Race (%)						<0.001
Mexican American	856 (13.18%)	128 (7.88%)	206 (12.69%)	263 (16.20%)	259 (15.95%)	
Other Hispanic	684 (10.53%)	121 (7.45%)	175 (10.78%)	197 (12.14%)	191 (11.76%)	
Non-Hispanic White	2575 (39.65%)	609 (37.50%)	622 (38.32%)	624 (38.45%)	720 (44.33%)	
Non-Hispanic Black	1481 (22.81%)	462 (28.45%)	357 (22.00%)	349 (21.50%)	313 (19.27%)	
Other races/ethnicity	898 (13.83%)	304 (18.72%)	263 (16.20%)	190 (11.71%)	141 (8.68%)	
Education level (%)						<0.001
Less than high school	1186 (18.26%)	192 (11.82%)	254 (15.65%)	331 (20.39%)	409 (25.18%)	
High school or GED	1474 (22.70%)	354 (21.80%)	330 (20.33%)	415 (25.57%)	375 (23.09%)	
Above high school	3834 (59.04%)	1078 (66.38%)	1039 (64.02%)	877 (54.04%)	840 (51.72%)	
The ratio of family income to poverty	2.57 ± 1.64	2.72 ± 1.69	2.73 ± 1.66	2.59 ± 1.62	2.24 ± 1.53	<0.001
BMI (kg/m^2^)	29.43 ± 7.14	24.89 ± 4.86	28.03 ± 5.44	30.54 ± 6.35	34.26 ± 7.94	<0.001
Energy intake (kcal)	2099.19 ± 850.07	2280.81 ± 918.99	2149.26 ± 829.47	2064.07 ± 849.48	1902.61 ± 749.82	<0.001
Protein intake (gm)	82.41 ± 37.04	90.22 ± 42.19	85.44 ± 35.94	80.06 ± 35.34	73.91 ± 31.96	<0.001
Carbohydrate intake (gm)	246.75 ± 108.10	267.66 ± 116.71	249.88 ± 105.64	244.67 ± 108.71	224.79 ± 96.07	<0.001
Dietary fiber intake (gm)	16.81 ± 9.54	17.66 ± 10.17	17.41 ± 9.54	17.03 ± 9.76	15.15 ± 8.41	<0.001
Total fat intake (gm)	82.18 ± 39.37	87.06 ± 41.43	84.03 ± 38.89	81.40 ± 39.84	76.25 ± 36.41	<0.001
LDL-C (mmol/L)	2.88 ± 0.92	2.70 ± 0.85	2.95 ± 0.89	2.97 ± 0.94	2.89 ± 0.97	<0.001
HDL-C (mmol/L)	1.39 ± 0.41	1.49 ± 0.41	1.40 ± 0.42	1.35 ± 0.41	1.34 ± 0.40	<0.001
TG (mmol/L)	1.25 ± 0.73	0.98 ± 0.62	1.23 ± 0.75	1.36 ± 0.74	1.42 ± 0.73	<0.001
TCHO (mmol/L)	4.84 ± 1.05	4.64 ± 0.97	4.91 ± 1.02	4.94 ± 1.08	4.89 ± 1.11	<0.001
Albumin, urine (mg/L)	45.97 ± 286.13	21.31 ± 121.14	27.86 ± 153.72	49.73 ± 313.02	84.97 ± 434.58	<0.001
Creatinine, urine (*μ*mol/L)	11662.59 ± 7160.83	12293.42 ± 7889.45	11850.50 ± 7033.55	11670.11 ± 7006.76	10836.46 ± 6578.52	<0.001
Albumin-creatinine ratio (mg/g)	41.61 ± 277.36	18.07 ± 130.60	26.88 ± 237.19	43.70 ± 286.36	77.77 ± 387.86	<0.001
HbA1c (%)	5.77 ± 1.15	5.39 ± 0.79	5.62 ± 1.00	5.85 ± 1.12	6.22 ± 1.42	<0.001
SBP (mmHg)	122.82 ± 17.70	117.03 ± 14.75	121.11 ± 16.51	125.19 ± 18.28	127.95 ± 19.01	<0.001
DBP (mmHg)	71.29 ± 12.37	69.29 ± 10.89	72.26 ± 11.57	72.54 ± 12.72	71.05 ± 13.84	<0.001
Heavy alcohol consumption (%)						0.003
Yes	1064 (16.38%)	243 (14.96%)	234 (14.42%)	292 (17.99%)	295 (18.17%)	
No	5430 (83.62%)	1381 (85.04%)	1389 (85.58%)	1331 (82.01%)	1329 (81.83%)	
Smoking status (%)						<0.001
Yes	3070 (47.27%)	681 (41.93%)	703 (43.31%)	833 (51.32%)	853 (52.52%)	
No	3424 (52.73%)	943 (58.07%)	920 (56.69%)	790 (48.68%)	771 (47.48%)	
Minutes sedentary activity (minute)	366.43 ± 202.35	359.24 ± 197.86	370.74 ± 205.89	355.52 ± 199.05	380.23 ± 205.71	0.002

Mean ± SD for continuous variables: the *P* value was calculated by the Kruskal–Wallis test. % for categorical variables: the *P* value was calculated by Fisher's exact test. WWI, weight-adjusted waist index; LDL-C, low-density lipoprotein cholesterol; HDL-C, high-density leptin cholesterol; TG, triglyceride; TCHO, total cholesterol; HbA1c, glycosylated hemoglobin; SBP, systolic blood pressure; DBP, diastolic blood pressure.

**Table 2:** Correlation between body WWI (cm/√ kg) and SUA (*μ*mol/L).

Exposure	Model 1		Model 2		Model 3	
	*β* (95% CI)	*P* value	*β* (95% CI)	*P* value	*β* (95% CI)	*P* value
WWI (cm/√ kg)	12.97 (10.53, 15.41)	<0.0001	25.38 (22.83, 27.94)	<0.0001	5.64 (2.62, 8.66)	0.0003
WWI categories						
Q1 (8.38–10.41)	Ref		Ref		Ref	
Q2 (10.41–10.98)	9.56 (3.79, 15.32)	0.0012	15.61 (10.41, 20.82)	<0.0001	−1.37 (−6.40, 3.66)	0.5925
Q3 (10.98–11.56)	22.47 (16.71, 28.23)	<0.0001	32.90 (27.44, 38.36)	<0.0001	5.40 (−0.17, 10.97)	0.0576
Q4 (11.56–14.14)	26.33 (20.57, 32.09)	<0.0001	49.20 (43.34, 55.06)	<0.0001	7.93 (1.42, 14.45)	0.0170
Subgroup analysis stratified by sex						
Male	18.38 (15.15, 21.62)	<0.0001	28.76 (24.75, 32.76)	<0.0001	8.16 (3.10, 13.23)	0.0016
Female	27.59 (24.62, 30.55)	<0.0001	24.27 (21.00, 27.54)	<0.0001	5.67 (1.96, 9.38)	0.0028
Subgroup analysis stratified by age						
≥20, <40	8.04 (3.98, 12.10)	0.0001	27.88 (24.46, 31.30)	<0.0001	7.05 (2.59, 11.51)	0.0020
≥40, <60	10.10 (5.41, 14.79)	<0.0001	25.01 (20.73, 29.29)	<0.0001	8.24 (3.34, 13.14)	0.0010
≥60, <80	12.42 (6.28, 18.56)	<0.0001	26.23 (20.20, 32.26)	<0.0001	6.77 (−0.08, 13.63)	0.0530
≥80	3.58 (−11.10, 18.25)	<0.0001	4.82 (−9.81, 19.44)	0.5195	−10.82 (−27.01, 5.37)	0.1920
Subgroup analysis stratified by race						
Mexican American	6.33 (−0.73, 13.40)	0.0791	24.62 (17.48, 31.77)	<0.0001	7.62 (−0.31, 15.56)	0.0600
Other Hispanic	14.34 (6.25, 22.43)	0.0005	19.46 (11.13, 27.79)	<0.0001	3.24 (−6.35, 12.83)	0.5083
Non-Hispanic White	16.34 (12.60, 20.09)	<0.0001	26.14 (22.24, 30.04)	<0.0001	2.21 (−2.44, 6.87)	0.3508
Non-Hispanic Black	18.07 (12.88, 23.26)	<0.0001	26.99 (21.55, 32.43)	<0.0001	7.26 (0.83, 13.70)	0.0272
Other race/ethnicity	6.49 (−0.53, 13.52)	0.0703	24.89 (17.56, 32.22)	<0.0001	11.88 (3.26, 20.51)	0.0071

Model 1: no covariates were adjusted. Model 2: age, sex, and race/ethnicity were adjusted. Model 3: age, sex, race/ethnicity, education level, the ratio of family income to poverty, BMI, energy intake, protein intake, carbohydrate intake, dietary fiber intake, total fat intake, LDL-C, HDL-C, TG, TCHO, urine albumin, urine creatinine, albumin creatinine ratio, HbA1c, SBP, DBP, heavy alcohol consumption, smoking status, and minutes sedentary activity were adjusted. In the subgroup analysis stratified by sex, age, and race/ethnicity, the model is not adjusted for sex, age, and race/ethnicity, respectively.

**Table 3:** Threshold effect analysis of WWI on SUA in males using the two-piecewise linear regression model.

SUA	Adjusted *β* (95% CI)	*P* values
Male		
Model 1: fitting model by standard linear regression	8.16 (3.10, 13.23)	0.0016
Model 2: fitting model by two-piecewise linear regression		
Inflection point		11.5
WWI <11.5(cm/√kg)	11.41 (5.62, 17.20)	0.0001
WWI >11.5(cm/√kg)	−5.09 (−17.63, 7.45)	0.4261
*P* for the log-likelihood ratio test		0.023

Age, race, education level, the ratio of family income to poverty, BMI, energy intake, protein intake, carbohydrate intake, dietary fiber intake, total fat intake, LDL-C, HDL-C, TG, TCHO, urine albumin, urine creatinine, urine albumin creatinine ratio, HbA1c, SBP, DBP, heavy alcohol consumption, smoking status, and minutes sedentary activity were adjusted.

**Table 4:** Threshold effect analysis of WWI on SUA in non-Hispanic White using the two-piecewise linear regression model.

SUA	Adjusted *β* (95% CI)	*P* values
Non-Hispanic White		
Model 1: fitting model by standard linear regression	2.21 (−2.44, 6.87)	0.3508
Model 2: fitting model by two-piecewise linear regression		
Inflection point		11.08
WWI <11.08(cm/√kg)	−7.08 (−14.39, 0.23)	0.0576
WWI >11.08(cm/√kg)	10.73 (3.78, 17.68)	0.0025
*P* for the log-likelihood ratio test		0.001

Age, sex, education level, the ratio of family income to poverty, BMI, energy intake, protein intake, carbohydrate intake, dietary fiber intake, total fat intake, LDL-C, HDL-C, TG, TCHO, urine albumin, urine creatinine, urine albumin creatinine ratio, HbA1c, SBP, DBP, heavy alcohol consumption, smoking status, and minutes sedentary activity were adjusted.

**Table 5:** Threshold effect analysis of WWI on SUA in other race/ethnicity using the two-piecewise linear regression model.

SUA	Adjusted *β* (95% CI)	*P* values
Other races/ethnicity		
Model 1: fitting model by standard linear regression	11.88 (3.26, 20.51)	0.0071
Model 2: fitting model by two-piecewise linear regression		
Inflection point		12.14
WWI <12.14(cm/√kg)	7.79 (−1.26, 16.84)	0.0918
WWI >12.14(cm/√kg)	87.10 (33.93, 140.27)	0.0014
*P* for the log-likelihood ratio test		0.004

Age, sex, education level, the ratio of family income to poverty, BMI, energy intake, protein intake, carbohydrate intake, dietary fiber intake, total fat intake, LDL-C, HDL-C, TG, TCHO, urine albumin, urine creatinine, urine albumin creatinine ratio, HbA1c, SBP, DBP, heavy alcohol consumption, smoking status, and minutes sedentary activity were adjusted.

## Data Availability

The data used to support the findings of this study are available from the corresponding author upon request.
